# How to optimise creative art therapy to foster the mental health of refugee adolescents? A Delphi study protocol

**DOI:** 10.1371/journal.pone.0308620

**Published:** 2024-10-16

**Authors:** Mohannad Ramadan, Ann Nolan, Kristin Hadfield, Tania Bosqui, Meg Ryan

**Affiliations:** 1 Trinity Centre for Global Health, Trinity College Dublin, Dublin, Ireland; 2 School of Medicine, Trinity College Dublin, Dublin, Ireland; 3 School of Medicine, Hashemite University, Zarqa, Jordan; 4 School of Social Work and Social Policy, Trinity College Dublin, Dublin, Ireland; 5 School of Psychology, Trinity College Dublin, Dublin, Ireland; 6 Department of Psychology, American University of Beirut, Beirut, Lebanon; Bangabandhu Sheikh Mujib Medical University (BSMMU), BANGLADESH

## Abstract

**Objective:**

This study aims to identify the most recommended components of creative art therapy (CAT) to improve the mental health of refugee adolescents.

**Design:**

A three-round Delphi design is proposed. The first round will include semi-structured interviews with a panel of 12 CAT professionals worldwide and 12 refugee adolescents aged 10–24 in Jordan with a history of participating in creative arts interventions. The hybrid approach of coding and thematic analysis will be conducted to develop statements on recommended CAT components from the interview narratives. In the second round, the same and newly enrolled 24 professionals and 24 refugee adolescents will be asked to rate the statements according to their importance, propose new statements, and add comments. A similar procedure will be followed in the third round, where panellists will rate new and old statements after perusing the feedback from the second round.

**Main outcome measure:**

A statement will gain consensus and indicate essential components when rated ‘essential’ or ‘very important’ by > = 80% of panellists. Very important components are those with the same ratings by 60–79.9% of panellists.

**Results:**

A list of essential and very important components, perspectives and suggestions will be provided to guide practice and intervention development.

## 1. Introduction

### 1.1 Background

Many voices in the literature infer the promising evidence of creative art therapy in improving the mental health of refugees. Generally, creative art therapy has been practised for a long time, but the growth of evidence on its benefits is not parallel with the increasing number of related publications [1]. Very few empirical articles examined creative art therapy intervention effectiveness in fostering the mental health of refugee adolescents and showed equivocal findings. Further, a lack of elaboration on design, implementation and adaptations was apparent. Refugee adolescents experience multifaceted challenges, necessitating resilient, interdisciplinary interventions that engage individuals, families, and communities to address their developmental needs and promote mental health [2, 3]. Also, according to Frounfelker et al. [4], research that integrates refugees into the design, delivery, and evaluation of interventions is likely to enhance the acceptability and effectiveness of mental health services. Another noted challenge to advancing the evidence base of creative art therapy is the heterogeneity in practice, which renders comparison between the interventions not possible [5].

### 1.2 Rationale of the study

To date, no studies have pursued optimising creative art therapy for refugees in adolescence. Thus, we framed a research question of ‘how to effectively design, implement, and adapt creative art therapy (CAT) interventions to foster refugee adolescents’ mental health?’. This study will provide qualitative and quantitative input from a panel of creative art professionals and refugee adolescents. This is a protocol for a pre-fixed three-round Delphi study. The first round will be semi-structured interviews with an expert panel of CAT professionals and refugee adolescents to develop statements on recommended CAT components. More panellists will be asked to rate and propose statements via two subsequent questionnaire-based rounds to obtain consensus, agreements and narrative feedback.

## 2. Methodology

### 2.1 Aims of the study

This study aims to delineate the most recommended design, approaches, activities, and adjustments of creative art therapy for refugee adolescents’ mental health from the perspective and experience of a panel of creative art professionals and refugee adolescents with creative arts experience in a three-round Delphi study design. The first round aims to generate statements on recommended creative art therapy intervention components by interviewing eligible panellists. To weigh and prioritise the intervention components, the same and newly enrolled panellists with the same eligibility criteria will be asked to rate the statements based on importance in the second and third rounds.

### 2.2 Terminology

Owing to the diversity of terminologies in the context of art therapy, it is necessary here to clarify the key concepts. In this protocol, creative art therapy implies any arts-based activity aimed at the mental health of adolescent refugees in the frame of promotion (prevention), therapy and trauma recovery. This study does not investigate CAT in psychological assessment as we assert it would demand additional or different methods. The authors chose creative art therapy to flag the creativity and expression ingredients of art therapy. In the absence of a standard concrete CAT intervention, the umbrella of the CAT concept in this research captures any interactive initiative that uses creative art mediums in the form of individualised or group activities, workshops, sessions, or programs. Creative arts embrace all art mediums, visual and body-movement-based. Visual arts imply crafting tactile artwork, like painting and sculpture, whereas body-movement-based arts indicate non-tactile artwork, like play theatre and dancing. This protocol only targets active participation in arts, so passive arts like listening to music are not at the core of this study and will not be included. Finally, we will use the term “refugee adolescents” to reflect the age group between 10–24 years following Sawyer et al. [6] definition and refer to them in the text as ‘adolescents’ to avoid the risk of intrusive and discriminatory inferences of the term ‘refugee adolescents’.

### 2.3 Developing the interview questions for the first round

As our study will include diverse intervention ingredients, including persons, place, process, and adaptations, we utilised the concept of ‘components’ to help us categorise the data we gleaned from the systematic literature review. These components represent intervention compartments in the logic of design, implementation, and adaptation. The design component points to the planned structure, while implementation tells the activities, approaches, and techniques within the intervention. The approach is the style, pattern, and strategy the therapist adopts to deliver the intervention activities meaningfully and efficiently. When the intervention is tailored to a specific context(s) without changing the core structure of the intervention, these are considered adjustments. If, on the other hand, the structure is subjected to significant modifications, then this is recognised as intervention adaptation. Implementation implies on-the-ground translation of components into practices to achieve the sought objectives. Mental health is “the mental well-being that enables people to cope with the stresses of life, realise their abilities, learn and work well, and contribute to their community” [7]. We then sorted the components into categories and ordered these categories according to the stages of the intervention, which are pre-, during, post-artmaking and ‘in all stages’. The categories and the chronological order aim to organise the interview questions and create the codes and themes for interview narrative analysis. The categories of creative art therapy practice components and a simple description of each category are shown in (Table 1).

**Table 1:** Categories of creative art therapy components at each intervention stage and a brief description of each category.

Category	Stage of intervention	Description
**Team & roles**	Pre-intervention	Characteristics, qualifications, and roles of therapy team personnel
**Structure**	Pre-intervention	The planned milestones or skeleton of the intervention
**Setting**	Pre-intervention	Characteristics of the place to carry out the activities
**Environment**	Pre-intervention	Factors surrounding the participants during practising arts
**Introduce**	Pre-intervention	Providing an overview of the intervention to the participants before launch
**Initiate**	Pre-intervention	Special activities to open and begin the intervention (e.g., icebreaking)
**Art mediums**	During intervention	What art medium(s) to be used (e.g., painting, dancing, mixed)
**Content**	During intervention	Topics, scenarios, and stories to be expressed through the artwork or performance
**Other Non-arts activities**	During intervention	Activities that are not based on arts (e.g., Breathing Exercises)
**Choice**	During intervention	The participants can choose the topic or go for individual sessions
**Flexibility**	During intervention	How much can the participants improvise and be spontaneous in doing the activities or be strict with the design of the intervention and therapists’ instructions?
**Relationship**	During intervention	Building relationships and friendships between the participants themselves or with the therapists
**Share, Express, Communicate**	During intervention	The way the participants share their thoughts through artworks or messages through art performance. The way they can express their ideas and feelings through the creative arts. The way they interact and socialise with other participants and with the therapists.
**Flow**	During intervention	How do the intervention sessions/activities run harmoniously to accomplish the best results?
**Dosing**	During intervention	Number, duration, and frequency of sessions
**Acceptability**	Through all the intervention	Making sure the activities are coherent, clear, easy, and not burdening the participants
**Trust/Safety**	Through all the intervention	How to build trust between all individuals in the intervention, create feelings of safety and ensure privacy?
**Engagement**	Through all the intervention	Keeping the participants wanting to continue, enjoying, excited and not bored.
**Assessment**	Through all the intervention	Techniques to examine the area of improvement in participants’ mental health
**Ending**	Post-intervention	Approach and activities to finalise the intervention and say Goodbye.
**long term outcomes**	Post-intervention	How can the things learned or the skills acquired in the intervention help the participants in their daily lives and even the future after the intervention?
**Age**	Adjustments	Adapting the design, approach, and practice to the age subgroups (early, middle, late adolescence) or another categorisation
**Gender**	Adjustments	Adapting the design, approach, and practice to the gender of the participants (i.e., males, females, non-binary gender)
**Culture**	Adjustments	Adapting the design, approach, and practice to the cultural norms of the participants (e.g., African or Islamic culture)

### 2.4 Research design

**Fig 1:**
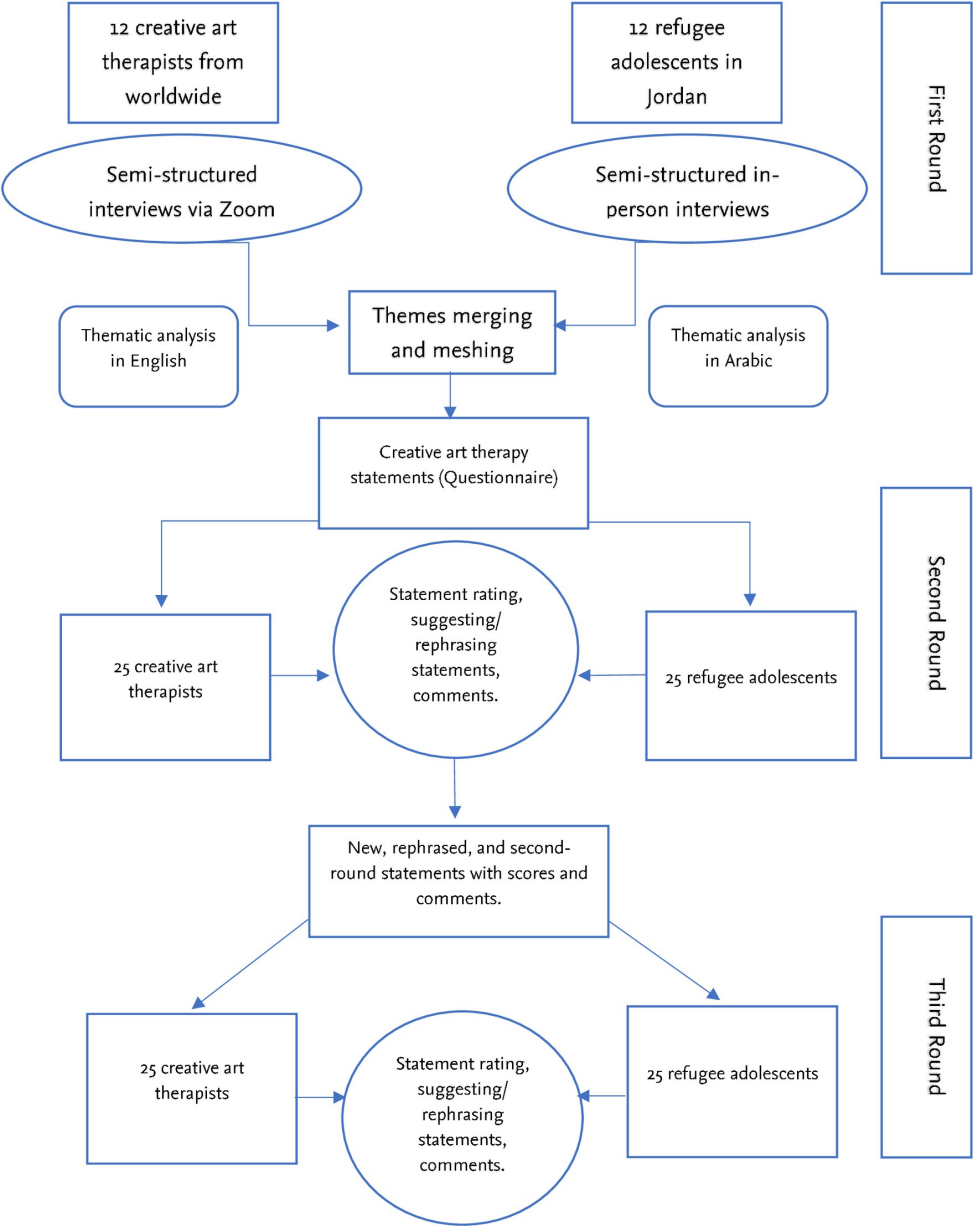
Design and the process of the Delphi study. Creative art therapy professionals and refugee adolescents will undergo semi-structured interviews in round 1 to generate statements on best practices. In the second and third rounds, the same and new participants following the same eligibility criteria will rate and discuss these statements via surveys.

The study will be conducted in a three-round Delphi design. Our sample will include a panel of two expert groups: creative art professionals and refugee adolescents. The sampling and study procedure will take different approaches, settings, and geographical scope for each group. As a result, we will demonstrate the methods separately for each group while referring to them as the “professional panel” and the “adolescent panel” as shown in **Fig 1**. The first round in our study consists of semi-structured interviews with the panel to generate statements. In the second round, the panellists will be requested to rate the statements using a self-filled questionnaire and openly provide feedback and perspectives. Further agreement will be sought in the third round, wherein statements, scores, and feedback from the second round will be shared with the panel of the third round. We will follow the Guidance for Conducting and Reporting Delphi Studies (CREDES) [8] and directions and advancements by Beiderbeck et al. [9] to underpin the implementation and reporting of the study.

### 2.5 Participants

Professional panel: The concept of professionals in this study is not exclusive to certified creative art therapists. Clinicians, researchers, artists, and art therapists would be eligible for the professional panel if they meet one or both eligibility criteria: 1- At least three years of experience working in creative art therapy with active participation in at least one creative arts intervention for refugee adolescents spanning the age range of 10–24. 2- Researchers with at least one peer-reviewed primary interventional study in the field of creative art therapy for refugee adolescents. Professional participants will be sought worldwide and from different professional backgrounds (e.g., medicine and health, academia and arts). We will use Expertscape (https://expertscape.com), a website that provides a platform for locating and identifying experts in various medical fields to find and contact potential therapists or researchers. We chose Sawyer et al.’s [6] age range of adolescence (i.e., 10–24) to be compatible with the systematic review that informed the conceptualisation of this study. The study frame for the professional group is not restricted to geography or time. Consequently, expert professionals from high- and low-middle-income countries (HIC and LMIC) who are eligible will be invited to take part. Each professional can participate in at least one round after being fully informed about the study methodology, participation rights and conditions. Adolescent panel: This group consists of refugee adolescents aged 10–24 of any origin and residing in Jordan who have experienced at least one creative arts workshop, program, or intervention. The adolescent group will be invited to participate through the affiliated organisations and community-based societies that delivered the intervention. Similarly, they can participate in at least one round following obtaining informed assent and consent from the adolescents and their caregivers or legal guardians, respectively. The implementation for this group will take place in Jordan, and the Principal Investigator will interview and survey the adolescents in person in the setting of the collaborating organisation or following the preference of the adolescents and their caregivers. The country of Jordan was chosen specifically for feasibility reasons as the principal investigator is Jordanian and has good access to several local and international organisations providing mental health care for refugees from several countries. Due to limited funds and resources, we will not be able to enrol refugee adolescents in other countries.

### 2.6 Sampling

Professional panel: We will adopt three steps of convenient sampling. First, we will send an invitation email to candidate professionals from the authors’ network, attaching the Invitation letter, study design flyer, participant information leaflet and the informed consent form. We will respond to inquiries sent back by the invited professionals and provide more elaboration where needed. Next, snowballing will be pursued by requesting the participants to recommend and liaise the study team with new potential candidates. The recommended candidates will be contacted through email to check their eligibility before sending the invitation email. The third step is to contact creative/art therapy associations and non-profit organisations, navigate social media profiles of potential candidates, and search bibliographies of relevant empirical peer-reviewed articles to reach candidate creative art therapy researchers. Adolescent panel: We will contact national or international organisations that implemented creative art therapy for refugee adolescents in Jordan. Upon agreement, the interviews will be conducted where convenient to the participants. Guardians of adolescents under 18 and adolescents aged 18–24 will be contacted directly with the support of the collaborating organisation. The principal investigator, a native Arabic speaker, will carry out phone calls with the guardians or the adolescents themselves and invite them to in-person meetings at the organisation’s centre or where preferred. In the meeting, the principal investigator will answer questions from the guardians about their child’s participation. The adolescent will be allowed to ask questions, and the principal investigator will ensure that all questions are explicitly answered. The guardian and the adolescent should clearly demonstrate the verbal agreement before signing the informed consent form by the guardian and the assent form by the adolescent. The sought sample size is 24 for the first round (professional panel = 12, adolescent panel = 12), which is above the minimum recommended sample size for initial interviews (i.e., five to eight panellists) in Delphi design [9, 10], and 50 for each of the second and third round (professional panel = 25, adolescent panel = 25). We benefited from Nasa et al. [11] research on appropriate Delphi methodology in optimising the volume of our sample. First, we assume good homogeneity within each panel group, which is attributed to our narrow eligibility criteria. Second, our research question can be answered by participants with specific and narrow criteria who are experts in our case (i.e., expert therapy professionals by ‘providing’ and expert adolescents by ‘receiving’ creative art therapy). Collectively, we assert that the sample size for each round is proper for our participants’ characteristics and research questions and, luckily, can be accommodated by our limited resources and timeline.

### 2.7 Pre-implementation piloting

We will conduct online interviews with a volunteering doctoral student at Trinity College Dublin to pilot the study implementation and anticipate technical roadblocks. The principal investigator will check the voice clarity in the pilot interview recordings and ensure proper storage. During the pilot interviews, the flow and consistency of the interview questions will be discussed with the research team, and the estimated time for the interview will be compared to the planned time and amended accordingly. Before launching the second round, the digital questionnaire will be piloted. Firstly, it will be shared with two Trinity graduate students, who will be asked to rate all the statements. Two more rounds of piloting will be carried out to check for any technological pitfalls. A similar piloting strategy will be followed before implementing each round for the adolescent panel. Pilot interviews will be conducted by the principal investigator with the psychologist—the field research assistant—from the collaborating organisation to ensure proper recording via the Dictaphone and establish the time needed to accomplish the interview. Before commencing the second round, the Microsoft Forms questionnaire will be technically tested by the principal investigator and the psychologist using a smart tablet and assessed for applicability, clarity, and content of the questionnaire. To ensure that the interview questions and the questionnaire statement are understandable, we will pilot them with a volunteering adolescent—aged 18–24, so guardian’s consent is not demanded—who is not necessarily of refugee background. The volunteering adolescent will discuss the questions and the statements with the principal investigator and provide suggestions for clarity and delivery. The volunteering adolescent’s recordings and questionnaires will be deleted immediately after the piloting.

### 2.8 Procedure

#### 2.8.1 Round 1

Professional panel: Online semi-structured interviews with 12 creative art professionals will be conducted in the first round. The interviews will be conducted via Zoom. A set of open-ended indicative questions, shown in Table 2, will be asked, allowing sufficient time for answers and discussions before moving to the next question. The panellists will be encouraged to share further remarks and raise any questions. The interview questions will be ordered through the chronological stages of creative art therapy as described above. Each question is phrased to reflect on one or more creative art therapy components. Each interview would expectedly last 60 minutes. After obtaining the interviewee’s permission, the interviews will be recorded to prevent data missing. Online interviews will be recorded through Zoom and stored in TCD-encrypted OneDrive. Adolescent panel: Adolescent refugees who consented to participate in the study will be interviewed face-to-face where convenient for them and their families. The interview will be recorded using a Dictaphone, and the interviewees will be informed that the recordings will be transcribed and deleted within one month—the same applied to the professional panel. The principal investigator will begin with an opening overview and icebreaking dialogue and then will ask and discuss the open questions and allow sufficient time for the young participants to share their ideas, perspectives, and experiences. We first phrased the questions for the adolescent panel in Arabic as simply as possible to meet their age and reflect the counterpart question for the professional panel before translating them into English for review by other research team members. As we anticipate a variety of creative art therapy experiences the adolescent panellists took part in, we will illustrate the concept of creative arts in the opening section and use the term “art workshop” in the questions to ensure better understanding by the adolescents. Most questions will be the same for the professional and the adolescent panels. Nonetheless, some questions do not apply to the adolescent panel, so the components of these questions will be only highlighted from the professionals’ side. Table 3 shows the interview questions for the adolescent panel in English.

**Table 2:** Interview questions per creative art therapy category for the professional panel.

	**Introduction: In this friendly interview, we would like to get your explicit answers**
	**to the following questions to define the features of creative art therapy intervention to**
	**improve the mental health of refugee adolescents. You are welcome to share**
	**your viewpoints and perceptions around any point in the questions.**
Category	***Pre-Intervention***
Team & roles	What are the requirements, skills, and roles of the therapy team?
Setting	What do you think makes a setting good and supportive for creative art therapy?
Environment	What is a suitable environment for creative art therapy intervention?
Initiate	How would you initiate the intervention? If presented, How do persons introduce themselves?
	***During Intervention***
Art mediums	What forms of arts and materials would you use in the intervention?
Content	What specific issues or themes do you encourage adolescents to explore and express through their artwork during the therapy?
Other activities	Besides creative arts, what non-arts activities do you recommend incorporating into the intervention to enhance the therapeutic experience?
Choice	What degree of Autonomy/ improvisation is good to offer to adolescents?
Flexibility	When do you allow adolescents to choose their activities? When do you think they can go to individual or group work?
Relationship	How would you build a good relationship with adolescents? How would you encourage good relationships between them?
Share/express/communicate	How would you encourage adolescents to express their creative work during the therapy sessions? What methods do you find effective for adolescents to communicate their thoughts and emotions through artwork? How do you facilitate sharing their artwork and thoughts, and with whom do you encourage them to share their creations during the therapeutic process?
Flow	How can we maintain a balanced and consistent flow of activities during the creative art therapy sessions?
Dosing	In your opinion, what is the number, duration, and frequency of the art therapy sessions for best results?
	***Through all the intervention***
Acceptability	How can you make the Intervention coherent? How can you make sure the participants are not burdened? *Theoretical Framework of Acceptability* (TFA) [18].
Trust/Safety	How can you establish a sense of trust and safety during the intervention?
Engagement	What strategies do you follow to enhance adolescent engagement throughout the creative art therapy process?
Assessment	How would you assess the progress and success of the intervention during and after implementation?
	***Post-intervention***
Ending	How would you close/finish the intervention?
long term effect	How can you make the adolescents take and apply what they learned in their daily lives?
	***Adjustments***
Age	What considerations would you take to make the intervention appropriate for ages 10 to 24 years?
Gender	How can the gender of participants impact the intervention’s design, and how would you adapt the intervention to address the specific gender profile?
Culture	When working with refugee adolescents, how do you consider and accommodate their cultural values and backgrounds during the therapy?

**Table 3:** Interview questions per creative art therapy category for the adolescent panel.

	**Ice breaking:** Can you tell me about yourself? What are your hobbies/interests?
	**Creative arts are those where you either make a piece of art like drawing, painting,**
	**sculpture, or writing or perform like singing, acting, or dancing. We know that arts**
	**are not attractive to everybody, but because you attended an art workshop in (..)/ by (..),**
	**you are our expert, so we would like to hear your ideas and opinions on how to improve**
	**it by asking you some questions.**
	لقاء تعارف: هل يمكنك أن تخبرنا قليلا عن نفسك؟ أخبرنا عن هواياتك, إهتماماتك.
	بداية دعنا نخبرك عن الفن الإبداعي, وهو القيام بصنع قطعة فنية مثل الرسم والنحت, أو القيام بأداء فني مثل الغناء, الرقص, أو التمثيل. نعلم جيدا أن الفنون لا تستهوي الجميع, لكن لكونك شاركت في ورشة علاج بالفن, نحن نعتبرك خبيرنا في هذا المجال. لذلك, نرغب بسماع أفكارك وآراءك لكيفية جعل هذه الورشة أفضل من خلال عدد من الأسئلة التفاعلية.
Category	***Pre-Intervention***
Opener/Structure	Hello! Let us start with a funny brainstorming! We would like you to use your imagination to describe the most exciting creative arts workshop you can think of. What does it look like?
	مرحبا! دعنا نبدأ بقليل من المرح والتفكير, نرغب في استخدام مخيلتك في وصف ورشة علاج بالفن ممتعة بالنسبة لك. كيف يمكن أن تصفها لنا؟
Team	What do you think are the important roles of the workshop supervisors?
	ما هي الأدوار المهمة للمشرفين على الورشة من وجهة نظرك؟
Roles	N/A
Setting & & Environment	What does the place in which you like to make/perform art look like?
	كيف يبدو لك المكان المناسب لاجراء هذه الورشة؟
Introduce	What do you think you need to know before participating in the workshop? What things if you know they will encourage you to participate?
	ماذا تعتقد انك تريد ان تعرف عن الورشة قبل المشاركة بها؟ ما هي الأشياء التي إذا علمتها قد تحفزك على المشاركة بها؟
Initiate	How do you like to start making artwork or performance?
	كيف ترغب أن تبدا صنع عملك الفني أو أداءك الفني؟ أو ما هي الخطوات الأولى لذلك؟
	***During Intervention***
Art medium	What art mediums or materials do you prefer?
	ما هو شكل أو أشكال الفن التي تفضل أن تختارها؟
Content	What do you want to talk about or tell through your artwork or performance?
	ما هل الأشياء التي تريد أن تتحدث عنها أو ترويها من خلال عملك الفني؟
Other activities	What other activities besides arts do you think would be exciting to do in the workshop?
	هل هناك أنشطة أخرى يمكن ان تفكر بها بجانب العمل أو الأداء الفني؟
Choice & flexibility	How do you prefer the activities in the workshop to be created or chosen?
	كيف تفضل أن يتم تصميم أو اختيار الأنشطة؟ من يمكن أن يقوم بذلك؟
Relationship	How can you make good relationships with others? What things can encourage you to interact with others in the workshop?
	كيف يمكن لك عمل علاقات أو صداقات جيدة مع المشاركين الاخرين؟ ماذا يمكن أن يشجعك على التفاعل مع الاخرين؟
Share/express communicate	When you create or perform art, how do you like to share it with others? and with whom would you feel comfortable sharing your work and thoughts?
	أثناء ممارستك للفنون, كيف تفضل أن تشارك عملك مع الاخرين؟ من هم الأشخاص الذين تشعر بارتياح إذا ما شاركت عملك معهم؟
Flow	N/A
Dosing	N/A
	***Throughout the intervention***
Acceptability	What do you feel about the workshop you took part in? (experienced)
	If you are invited to another creative arts workshop, what do you think can make it more
	appropriate for you? (anticipated).
	*Theoretical Framework of Acceptability (TFA)* [18].
	كيف تشعر أن الورشة التي شاركت بها كانت مناسبة لك؟ إذا تمت دعوتك للمشاركة في ورشة أخرى, ما هي الاشياء التي تعتقد انها قد تجعل الورشة مناسبة أكثر لك؟
Trust/Safety	What can make you trust the facilitators and other participants? How can you make them trust you? What makes you feel safe in the workshop?
	ماذا يمكن أن يجعلك تثق بالاخرين في الورشة؟ كيف يمكن أن تجعلهم يثقون بهم؟ ماذا يمكن أن يجعلك تحس بالأمان خلال المشاركة في الورشة؟
Engagement	What can make you excited and want to continue? What can make you bored or not want to continue?
	ما الأشياء التي تعتقد انها قد تجعلك منغمسا في الورشة؟ ما الأشياء التي قد تجعلك ضجرا ولا تريد الاستمرار في الورشة؟
Monitoring	N/A
	***Post-intervention***
Assessment	How do you think participating in a creative arts workshop could positively impact your life?
	كيف تعتقد أن المشاركة في ورشة العالج بالفن يمكن أن ثؤثر إيجابا في حياتك؟
Follow-up	N/A
	***Adjustments***
Age/gender/background	Can you share with me the kind of peers you would enjoy having with you while participating in the workshop’s activities?
	كيف يمكن أن تصف الأقران الذين قد تستمتع بمشاركة عملك الفني معهم؟
Culture	How does your culture influence your artwork or performance? How does the culture in Jordan influence your work?
	كيف يمكن أن تظهر ثقافة وطنك من خلال عملك أو أداءك الفني؟ كيف يمكن ان ان تظهر ثقافة الأردن في عملك أو أداءك الفني؟
Language	N/A

#### 2.8.2 Round 2

Professional panel: The questionnaire will present a list of statements on recommended creative art therapy design, activities, approaches, and adjustments developed from the interview narratives of the first round. The statements will be phrased by the principal investigator with assistance from the co-authors. The survey questionnaire will be distributed online via TCD-encrypted Microsoft Forms to 25 panellists, including both panellists from the first round and new panellists following the same enrolment process and eligibility criteria. The statements will be ordered in the questionnaire following the chronological stages of creative art therapy; pre-, during, and post-artmaking, and “through all stages”, in addition to the last section reflecting the special adaptations and adjustments. Adolescent panel: The transcripts of the interviews with the adolescents in the first round will be analysed to phrase statements in Arabic. The statement will be translated into English for review before being shared with the professional panel in the second round. In the second and third rounds, the adolescent panellists will use a TCD-encrypted Microsoft Forms questionnaire via a smart tablet. The statements generated from the components only applicable to the professional panel to report on will be shared with the adolescents if we see that the adolescents can sensibly rate them—this will be determined through discussion with the research team. Both groups will be asked to rate each statement using a four-point Likert scale: (1) essential, (2) very important, (3) slightly important, (4) not important, (5) cannot tell. Against each statement, a blank box will be available to the panellist to add comments concerning the statement. Additionally, we added a discussion box at the end of the questionnaire, enabling the panellists to share different thoughts or suggestions.

#### 2.8.3 Round 3

Round three will go in the same way as the second round. The third round aims to share information between the panellists to have stronger phrasing of the statements, generate new statements, and obtain consensus through “controlled feedback” [11], which is a classic approach in Delphi design. In our study, quantitative results represented by the statements’ scores in the second round, accompanied by qualitative data provided by the panellists through comments on statements—that are more expected when the panellist chose extreme answers—will be moved to the third round. Panellists will peruse the statement’s scores and comments and will be asked to rerate the statement, with or without adding another comment or proposing new statements. As this is a fixed three-round Delphi study, the statements proposed through the controlled feedback approach in this round will only be discussed in the results section and will not be subjected to further rating. At this stage, quantitative and qualitative feedback from the second round will be two-way shared with both the professional and the adolescent groups. We believe that information cross-sharing will set a dialogue between the providers and the recipients. This approach may provoke a bilateral understanding and mutual grasp of creative art therapy components.

### 2.9 Closing criteria

Delphi studies can be conducted in multiple rounds until targeted consensus is obtained, or the number of rounds can be planned in advance, regardless of the degree of consensus that will be reached [12]. We chose a prefixed number of rounds without seeking a degree of consensus to end the study. Modified Delphi is designed in two to three rounds planned in priori, in which the researchers usually craft statements from literature review and struggle to reach a consensus starting from the first round. This urgency in obtaining consensus can predispose to bias [11]. Accordingly, we prefer to preserve the validity of results rather than having an unstable consensus [11]. We are also keen to minimise the active participation of the research team in achieving consensus to minimise researchers’ bias, especially since this study’s aims do not urge for consensus. Although our Delphi is with prefixed three rounds, we advantage our methodology by using semi-structured interviews in the first round to generate data from the panellists themselves. The findings of our systematic literature review were used to create the categories and define components of the practice of creative art therapy. However, the panellists will be an equal source of conceptualisation through the interviews in the first round and the controlled feedback in the second and third rounds. We assert that the overall design of the study satisfies our aims to expand the volume of knowledge about creative art therapy practice and provide depth by measuring the essentiality and importance of therapeutic components.

### 2.10 Translation team and process in the adolescent group

This section will explicitly and transparently demonstrate the translation process needed in the adolescent group following the framework proposed by Abfalter et al. [13]. To increase rigour, the translation process will be evaluated via methodological recommendations for cross-language qualitative research [14]. As the adolescent panel will consist of adolescent refugees in Jordan, we will need to translate between English, which is the language of this study design and the result reporting at the end of the study, and Arabic, which is the language of implementation, and data collection and analysis for the adolescent panel in the three rounds. Even though some adolescent refugees can read and understand English, Arabic will be the sole language used in implementation for optimum standardisation. The translation process will commence at the stage of phrasing the semi-structured interview questions in Arabic and translating them into English for review by the study team. After conducting the interviews, the collected data will be analysed in Arabic, and the analysis results will be translated from Arabic to English to be combined with the results of the professional group interviews. The same team will translate statements from the second and third rounds. The translation team consists of the principal investigator, a Jordanian physician with experience in the healthcare field for refugee communities in Jordan, and a second translator. The second translator will be a bilingual Jordanian psychologist living in Jordan with proficient experience in mental health in low-setting areas. Both translators are bilingual (Arabic and English) and culturally close to the culture of adolescent panellists, which consequently would underpin the validity of the translation as per Al-Amer et al. [15]. Together, the first and the second translators will be responsible for translation through the implementation. They will manually translate the digital documents, including the interview transcripts and questionnaire statements. Discussion of discrepancies between the translation team will aim to obtain agreement and optimise the reliability of the translation. The statements will be translated between Arabic and English as the statements will be cross-shared between the two-panel groups. Committing to our terms with the participants, we will need to translate the results into Arabic only if the participants or their guardians ask for a brief of the results. Nonetheless, the results of the study will be displayed only in English.

### 2.11 Ethical considerations

Ethical approval for the professional panel was granted by the School of Medicine research ethics committee (Application No: 2442). The ethical approval for the adolescent panel was obtained from the Faculty of Health Sciences research ethical committee at Trinity College Dublin. The institutional review board of the School of Medicine of Hashemite University approved the fieldwork in Jordan (No.29/1/2023/2024). Data privacy and confidentiality for panellists comply with the European Union’s (EU) General Data Protection Regulations (GDPR). The informed consent form and the participation information leaflet will provide explicit elaboration on handling personal data and participation rights. Minor panellists—aged 10–17—will have to sign the assent form, and their caregiver/legal guardian will have to sign the informed consent form.

### 2.12 Data analysis

#### 2.12.1 Sociodemographic

We will collect minimal sociodemographic data from both groups. The aim of collecting these data is to picture the characteristics of the participants. The socio-demographic data will not be connected to the results of any round because assessing relationships between participants’ characteristics and their output is not within the objectives of this study. Upon participating in any round, the following sociodemographic data will be collected from the professionals: gender, age, nationality, number and locations of involvement in creative art therapy, qualification, years of experience in the leading career, and experience in the field of creative art therapy, and the characteristics of the adolescent refugees they worked with, comprising their age, gender, country of origin and settlement. For adolescent panellists, age, gender, country of origin, years since migrated, living place (only whether in a camp or urban setting), date and type of last CAT workshop, and number of CAT participations will be collected.

#### 2.12.2 First round

Aiming at rigour in our data analysis, we will adopt a hybrid approach of inductive and deductive coding and thematic analysis [16]. Inductive data coding utilises the narratives of qualitative interviews to sort the data without relying on pre-determined codes. In contrast, deductive coding draws on set-in-advance codes and categories. In this study, we will begin with deductive coding using the pre-defined categories we gleaned from our systematic literature review and the intervention stages we illustrated in the conceptualisation of this protocol. The categories shown in (Table 1) will be used to code the collected raw data from the semi-structured interviews. After that, the coded data will be fitted under three themes of intervention components: design, implementation, and adaptations. We will order the data according to the chronological stages of the intervention—pre-artmaking, during, post-artmaking, and through all—to tidy up and support the flow of the statements in the second round. Simultaneous with the Deductive (Top-Down) approach, we will integrate an Inductive (Bottom-up) approach [17] to capture additional codes and create new themes from the data provided by the panellists. The net objective of the thematic analysis is to structure statements on acceptable, engaging, effective, and feasible components of an intervention for refugee adolescents’ mental health. We will export the first-round data to the TCD-supported NVivo software version, through which we will conduct coding and thematic analysis.

#### 2.12.3 Consensus (second and third round)

Statements rated “essential” or “very important” by more than or equal to 80% of the panellists will win consensus and be classified as essential components. Statements rated less than 80% will be moved to the third round for further rating through the controlled feedback approach. Panellists of the third round will peruse the statements, their scores, and comments before rating again. New statements generated in the second round will be subjected to a one-time rating in the third round. By the end of the third round, statements that obtained consensus will be classified as essential components. At the same time, statements rated ‘essential’ or ‘very important’ by 60–79.9% of panellists will be classified as very important. The rest of the statements will be demonstrated against their scores, the number of rating rounds (one or two) they went through, and the number of panellists from each group (i.e., professionals and adolescents) who rated them. The Kappa coefficient will be calculated to test the internal consistency of the questionnaire in the second and third rounds. The quantitative consensus and agreement analysis will be conducted using Statistical Package for Social Sciences (SPSS).

## 3. Expected results

Our mixed methods study design is unique as it allows the exchange of viewpoints, perspectives, and experiences between the therapy providers (i.e., therapists and researchers) and the recipients (i.e., refugee adolescents). We expect to dive deep into impactful CAT practice by interviewing experts, and identify strongly recommended practice components through statement rating and controlled feedback.

## 4. Discussion

This article is novel as the first protocol for a Delphi study depicting recommended CAT practices to foster the mental health of refugee adolescents. Besides, the methods encompass semi-structured interviews with expert providers and expert adolescents. The CAT professionals’ panel will likely offer more generalisability of the outcomes because participants will be recruited from different countries with diverse experiences worldwide. In contrast, the adolescent panel would mainly include Syrian, Palestinian, and potentially Iraqi or Yemeni adolescent refugees. Thus, the restricted background of this group tends to be a limitation of this study. Implementing this protocol in different locations, settings, and contexts can provide a fuller picture of effective CAT components in improving the mental health of refugee adolescents.
